# AgCl-doped CdSe quantum dots with near-IR photoluminescence

**DOI:** 10.3762/bjnano.8.117

**Published:** 2017-05-29

**Authors:** Pavel Aleksandrovich Kotin, Sergey Sergeevich Bubenov, Natalia Evgenievna Mordvinova, Sergey Gennadievich Dorofeev

**Affiliations:** 1 Department of Chemistry, Lomonosov Moscow State University, 1 building 3 Leninskie Gory, Moscow 119991, Russia; 2 Laboratoire CRISMAT, UMR6508, CNRS-ENSICAEN, 6 boulevard Marechal Juin, Caen 14050, France

**Keywords:** Ag doping, CdSe quantum dots, doped semiconductor nanocrystals, IR photoluminescence, tetrapods

## Abstract

We report the synthesis of colloidal CdSe quantum dots doped with a novel Ag precursor: AgCl. The addition of AgCl causes dramatic changes in the morphology of synthesized nanocrystals from spherical nanoparticles to tetrapods and finally to large ellipsoidal nanoparticles. Ellipsoidal nanoparticles possess an intensive near-IR photoluminescence ranging up to 0.9 eV (ca. 1400 nm). In this article, we explain the reasons for the formation of the ellipsoidal nanoparticles as well as the peculiarities of the process. The structure, Ag content, and optical properties of quantum dots are also investigated. The optimal conditions for maximizing both the reaction yield and IR photoluminescence quantum yield are found.

## Introduction

Colloidal quantum dots (QDs) have attracted considerable attention because of their physical and chemical properties. These properties are dissimilar to those of bulk objects because of the small size of the QDs (a few nanometers) [1–3]. QDs can emit at arbitrary emission wavelengths [4–9] and this property is widely applied in many optical devices. CdSe QDs deserve special attention because of their bright photoluminescence (PL), photostability, and a considerable number of methods for their synthesis.

For many of the practical applications of QDs infrared PL (IR-PL) is very important: for the fabrication of optical fibers, lasers, biolabels and for the sensibilization of Si-solar cells. But the optical properties of CdSe QDs are limited by the band-gap energy of the bulk material (*E*_g_ = 1.74 eV) [10–11], which makes exciton IR-PL impossible. Therefore, the development of new synthetic methods for CdSe QDs with non-excitonic IR-PL is particularly relevant [12].

The most common way to obtain IR-PL in CdSe QDs is to add some optically active defects that reduce the energy of electron–hole recombination by trapping [13–14]. Charge carriers could be trapped by both surface defects [15–16] and volume defects [17–21]. It is known that doping CdSe QDs with Ag leads to the emergence of a low-energy band [22–23]. However, there are only a few publications devoted to Ag-doped CdSe QDs. The authors [23] used a cation-exchange method [24] to produce Ag-doped CdSe QDs with a low-energy band. However, this band becomes prominent only at 10 K. Our previous work proposed an oleate-based method (the in situ colloidal synthesis) to synthesize Ag-doped CdSe QDs with a wide band in the near-IR region along with an exciton band. In addition, a low-energy band could be clearly observed even at room temperature [22]. However, for Ag-doped CdSe NPs the PL does not extend into the energy range below 1.3 eV [22–23].

The major differences from our previous investigations are the type of Ag precursor and the way of adding this precursor to the reaction mass [22,25]. We used a silver complex of AgCl with triphenylphosphine (Ag_4_Cl_4_(PPh_3_)_4_) before [22], which should be more stable than our new precursor, namely a AgCl solution in trioctylphosphine (TOP). Ag_4_Cl_4_(PPh_3_)_4_ was added to the reaction mass in solid state without prior dissolution. There were some problems with setting the exact Ag to Cd ratio and with achieving a high concentration of silver and chloride ions in the reaction mass due to the restricted solubility of this complex.

In this work, we use a modification of the oleate method to prepare Ag-doped CdSe QDs using AgCl as a precursor of Ag. We focus our attention on the two origins of trap states: halides and silver. Halide ions have their own hole traps [26], which can reduce the energy of PL. They also play an important role in the surface passivation, which can lead to higher QYs and result in anisotropic growth of nanoparticles [27]. Herein, we explore the combined influence of both Ag and Cl ions in the modified oleate method on optical properties of CdSe QDs and find optimal conditions to obtain QDs with IR-PL. We also investigate structure, composition and shape of NCs obtained with the use of X-ray diffraction (XRD), transmission electron microscopy (TEM), and X-ray fluorescence spectroscopy (XRF).

## Results and Discussion

Each synthesis was identical but for the AgCl content (see Table 1). Specimen designation was as follows: AgCl_*N*, where *N*/32 is the molar ratio of Ag to Cd during synthesis.

**Table 1:** Amount of Ag and Cd precursors in synthesis.

	amount of precursor added		Ag to Cd ratio
	AgCl, μmol	Cd(oleate)_2_, μmol	

AgCl_0	0	500	0:32
AgCl_1	16	500	1:32
AgCl_2	31	500	2:32
AgCl_4	63	500	4:32
AgCl_8	125	500	8:32
AgCl_10	156	500	10:32
AgCl_12	188	500	12:32
AgCl_16	250	500	16:32
AgCl_24	375	500	24:32
AgCl_32	500	500	32:32
AgCl_40	625	500	40:32

### XRD and TEM investigations

**Figure 1:**
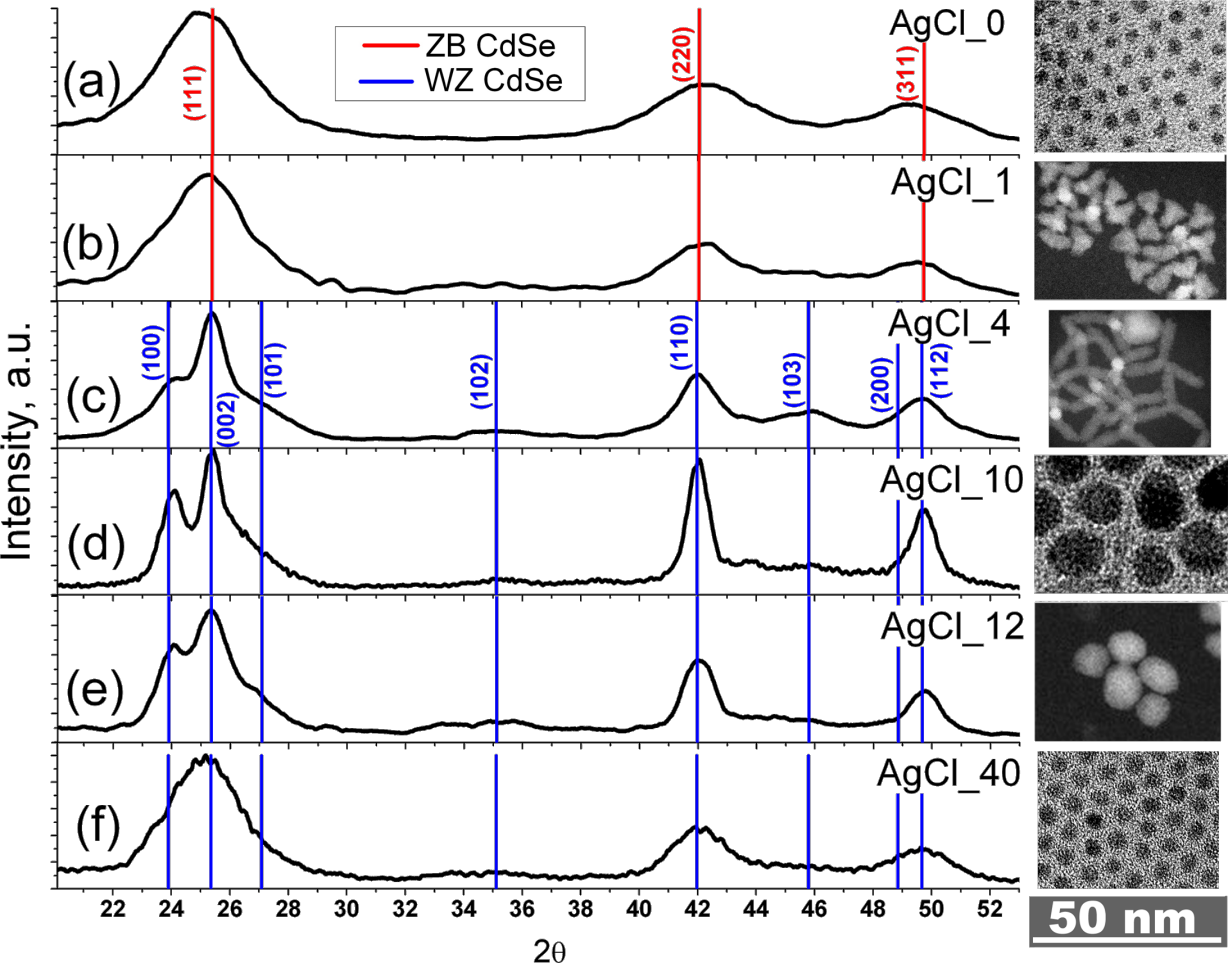
XRD patterns and TEM images of different samples with varying AgCl amount: (a) AgCl_0, (b) AgCl_1, (c) AgCl_4, (d) AgCl_10, (e) AgCl_12 and (f) AgCl_40. (ZB: zinc blende, WZ: wurtzite.)

Transmission electron microscopy (TEM) and X-ray diffraction (XRD) investigations of the obtained QDs are given in Figure 1. The structure and morphology of synthesized QDs depend on the amount of AgCl added during synthesis. The XRD pattern of undoped CdSe NPs (AgCl_0) (Figure 1a) contains three main peaks at diffraction angles of 2θ = 25.05°, 42.29° and 49.26° corresponding to (111), (220) and (311) of the cubic zinc blende (ZB) structure (*F*−43*m*, space group 216, *a* = 0.6077 nm (PC-PDF 19-191)). The NPs exhibit a nearly spherical shape with an average crystalline size estimated to be around 3 nm. The XRD peaks are broad due to the small size of the NPs.

**Figure 2:**
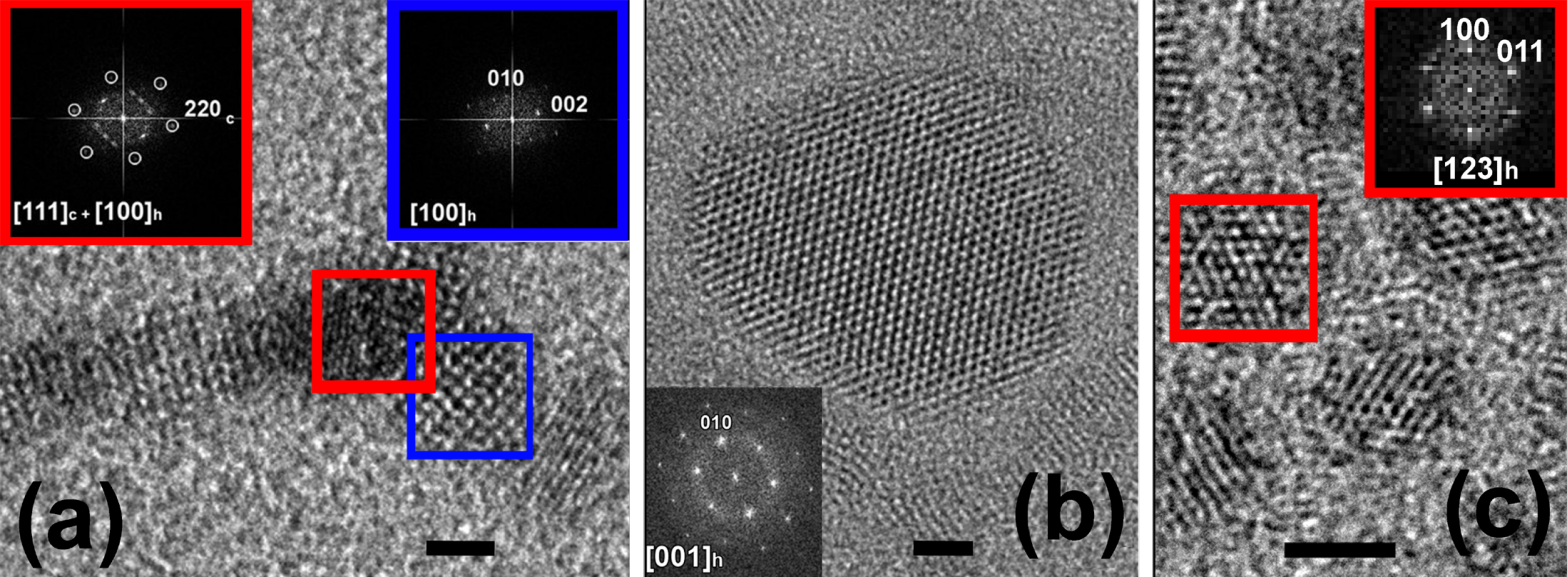
HRTEM images of TP (sample AgCl_8) (a), EPs of samples AgCl_10 (b) and AgCl_ 40 (c) with Fourier transform (FT) patterns of the TP core (left red FT), TP leg (blue FT) and of the EPs. Scale bar is 3 nm.

The addition of a small amount of AgCl results in the formation of anisotropic particles with tetrapod-like shape and XRD patterns that are still characteristic of cubic ZB structure (Figure 1b). Further increase of AgCl content in synthesis causes the formation of tetrapods (TPs) in samples AgCl_4 (Figure 1c) and AgCl_8 and the appearance of additional peaks in the XRD patterns. These additional XRD peaks can be attributed to the appearance of the hexagonal wurtzite (WZ) structure (*P*6_3_*mc*, space group 186 *a* = 0.4299 nm and *c* = 0.7010 nm (PC-PDF 8-459)). This structure appears in the legs of the TPs as is evident from high-resolution TEM (HRTEM) image (Figure 2a). In previous investigations similar CdSe TPs exhibited all WZ reflexes in their XRD pattern [27].

TEM investigation of the samples also revealed the presence of large NPs with sizes of tens of nanometres and irregular shape, i.e., large ellipsoidal particles (EPs) (see Figure 1 and Supporting Information File 1, Figure S1). For samples AgCl_1 to AgCl_10 with the increasing amount of added AgCl during synthesis leads to an increase of fraction and size of EPs (Table 2). Beginning with sample AgCl_12 EPs are the only product and TPs completely disappear. Hence, we called the range of AgCl concentration between samples AgCl_10 and AgCl_12 the “breaking point” (BP). A further increase of the amount of AgCl results in a decreasing size of the EPs (see Figure 1e,f, Table 2 and Supporting Information File 1, Figure S1). This effect is due to the decrease of the nucleation energy in the presence of more AgCl precursor during synthesis.

**Table 2:** Results of the examination of TEM images: fraction of TPs and EPs in the samples, characteristic particle sizes and NP volumes. Sizes of the QDs were found from TEM images as maxima of the size distributions. The error of size determination is about 1 nm (for the 1 to 5 nm sizes) and about 2 nm (for the 5 to 20 nm sizes).

sample	tetrapods (TPs)				spherical and ellipsoidal particles (EPs)		
	fraction in product, %	length of legs, nm	width of legs, nm	TP mean volume, nm^3^	fraction in product, %	average diameter, nm	EP mean volume, nm^3^

AgCl_0	0	—	—	—	100	3.0	14
AgCl_1	98	4.0	3.4	145	2	12.3	974
AgCl_2	97	6.5	3.8	195	3	13.0	1150
AgCl_4	94	9.5	3.4	345	6	12.7	1073
AgCl_8	92	11.0	3.8	499	8	13.5	1288
AgCl_10	5	17.0	3.7	731	92	15.0	1767
AgCl_12	0	—	—	—	100	9.5	449
AgCl_16	0	—	—	—	100	8.3	299
AgCl_24	0	—	—	—	100	6.9	172
AgCl_32	0	—	—	—	100	4.7	54
AgCl_40	0	—	—	—	100	4.0	34

Bearing in mind the large size and fraction of EPs in the samples (Table 2), we attributed the rather narrow and intense diffraction peaks of sample AgCl_10 to the hexagonal WZ structure of these particles (Figure 1d). HRTEM images of these particles and the corresponding FT patterns (Figure 2b) confirm this assumption. In samples AgCl_12 to AgCl_40, the WZ reflections become broader because the size of EPs decreases (Table 2 and Figure 1e,f). The structure of AgCl_40 was also confirmed by HRTEM (Figure 2c).

**Figure 3:**
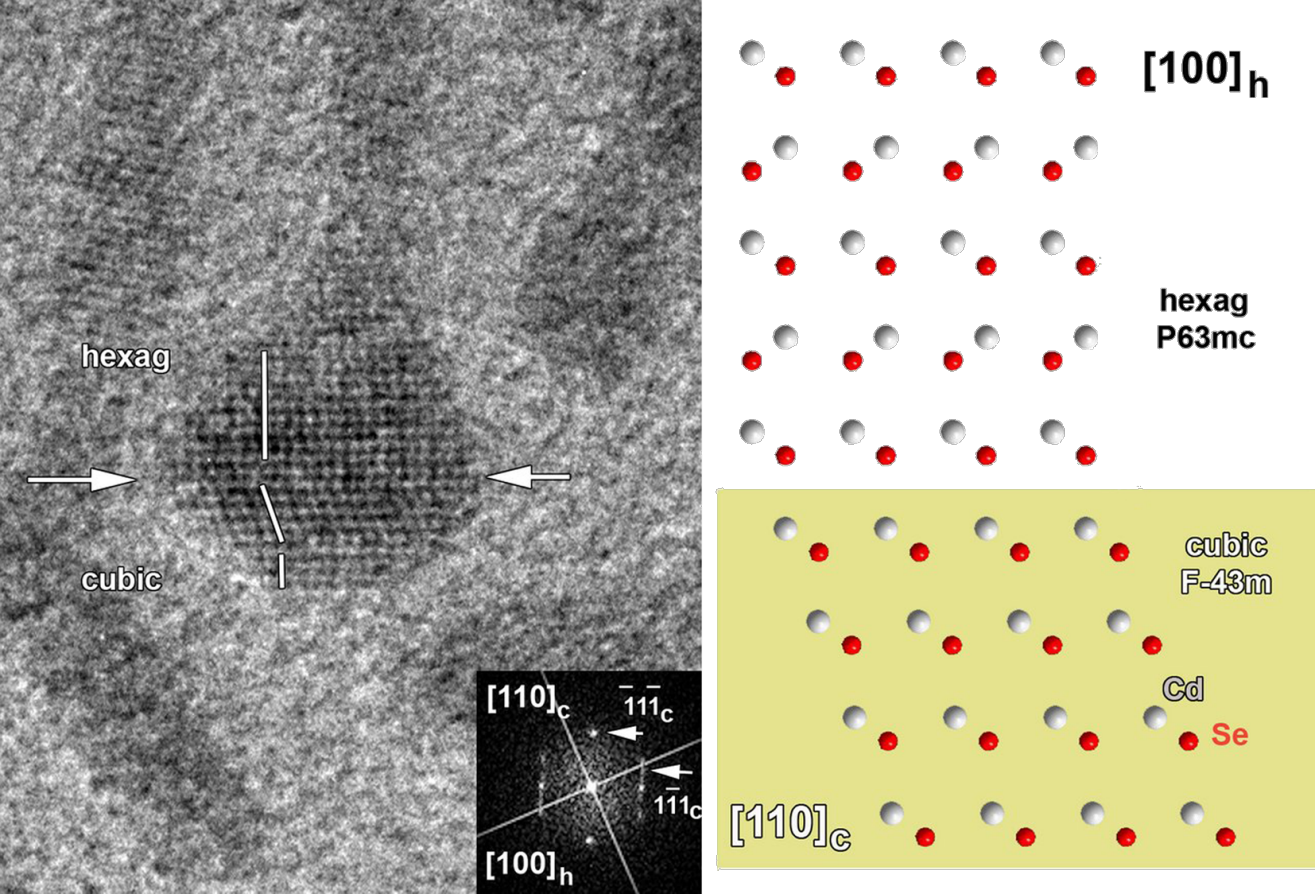
HRTEM image of the “match head” particle of sample AgCl_10 and the result of structure investigation with the use of FT.

In order to investigate the origin of EPs we analysed the intermediate sample AgCl_10. In this sample a small amount (2–3% of the total amount) of the so-called “match head” particles are present, and we propose that they are the intermediate form between TPs and EPs. HRTEM (Figure 3) showed that these particles are diphasic with gradual transitions of ZB to WZ in the core and, subsequently, in the legs. Based on this, we assume that large EPs are formed from legs by accretion on a core. It is clearly seen that the speed of that process increases with increasing amounts of AgCl during synthesis. There is a number of different mechanisms that could explain this process including dissolution of TPs, melting of TPs, Ostwald ripening and, which seems the most probable, their combinations. Some of these processes would lead to highly defective structures.

### XRF analysis and EDX/TEM imaging

During storage of the samples a mixture of Ag and Ag_2_Se was formed (see the XRD pattern in Supporting Information File 1, Figure S2). We investigated the silver content in the QDs over time using XRF. The amount of silver was found with the use of the calibration curve presented in the Supplementary data of [22]. The analytical signal was determined as the integral intensity ratio between the Ag Kα line (*S*_Ag_) and the Cd Kα line (*S*_Cd_). The calibration curve is described by the linear equation [22]:

[1]
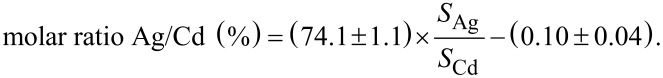


**Figure 4:**
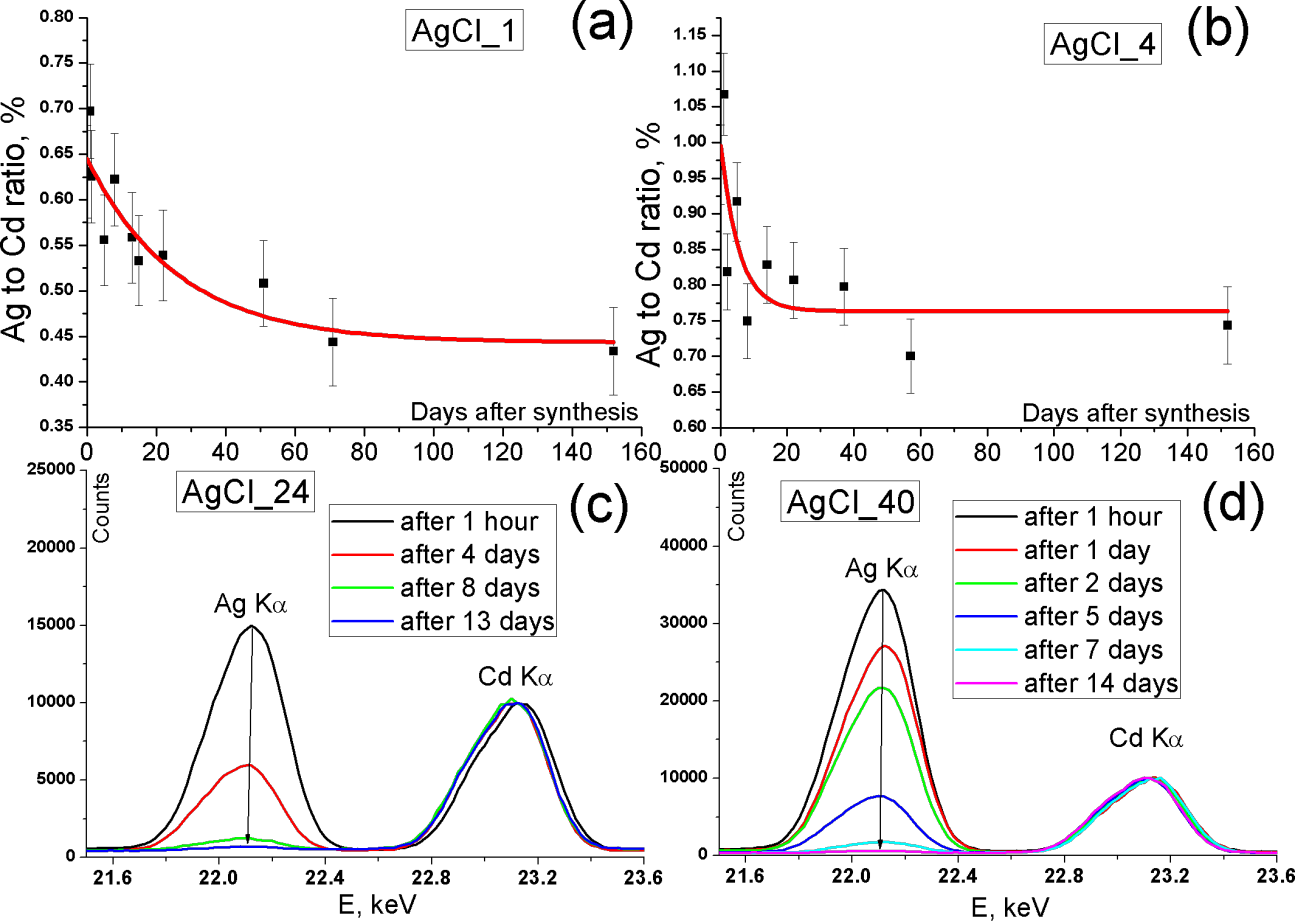
XRF analysis of Ag content during aging of NPs. (a,b) Results obtained by using Equation 1 for samples AgCl_1 and AgCl_4, respectively. (c,d) Evolution of XRF spectra of samples AgCl_24 and AgCl_40 over time.

Equation 1 is valid for the determination of Ag at concentrations up to 4 mol % Ag (to Cd). The samples were centrifuged before each measurement (21000*g*, 30 s) to remove suspended Ag. The first measurement was performed one hour after the synthesis. The amount of Ag decreased in all samples over time. The results of Ag content determined using Equation 1 are presented in Figure 4a,b (for the samples AgCl_1 and AgCl_4, respectively). Here we use an exponential law to interpret the experimental data.

The samples AgCl_24 and AgCl_40 had a much greater Ag amount that significantly exceeded 4 mol % Ag (to Cd) but the tendency also remains. Changes in XRF spectra of samples AgCl_24 and AgCl_40 with time are illustrated in Figure 4c,d.

**Figure 5:**
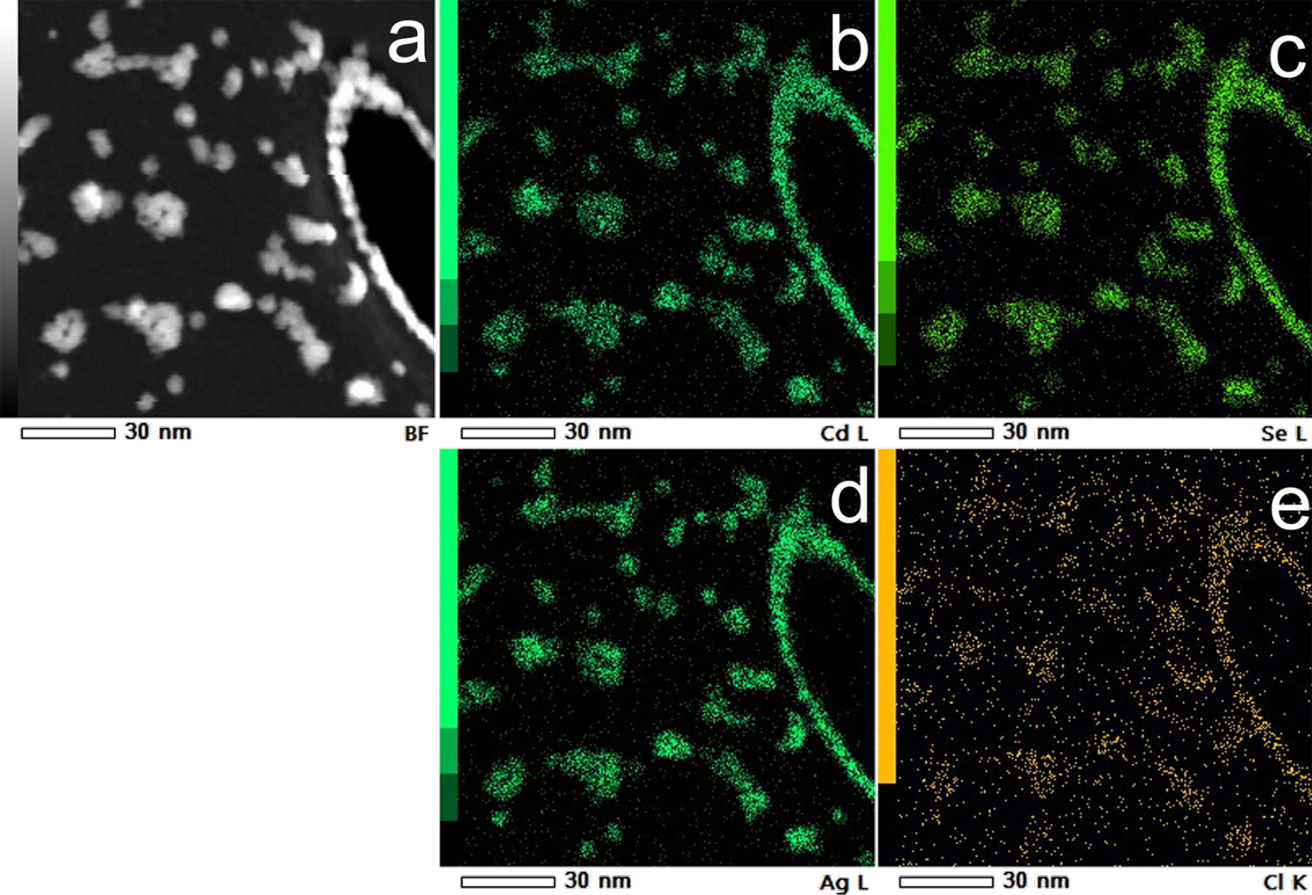
Element distribution maps of sample AgCl_32 on the day of the synthesis of NPs. (a) HAADF-STEM image, (b–e) positions of Cd, Se, Ag and Cl atoms respectively.

To investigate the location of Ag and Cl in NPs, element distribution maps with the use of energy dispersion X-ray (EDX) spectroscopy were obtained (Figure 5). Figure 5a shows a high angle annular dark field (HAADF) image of sample AgCl_32, which was obtained with the use of scanning transmission electron microscopy (STEM). Figure 5–e display the position of Cd, Se, Ag and Cl atoms. The investigation was made on the day of the synthesis of NPs. The colour brightness of one selected pixel is determined by the intensity of L (Cd, Se and Ag) or K (Cl) line in EDX spectrum. The element distribution maps show that Ag is present in QDs right after the synthesis. The weakness of the Cl signal does not allow one to precisely determine the position of Cl atoms, but the repetition of the outlines of the NPs allows for the assumption that chlorine is likely located inside the QDs.

### PL, absorbance and QY measurements

**Figure 6:**
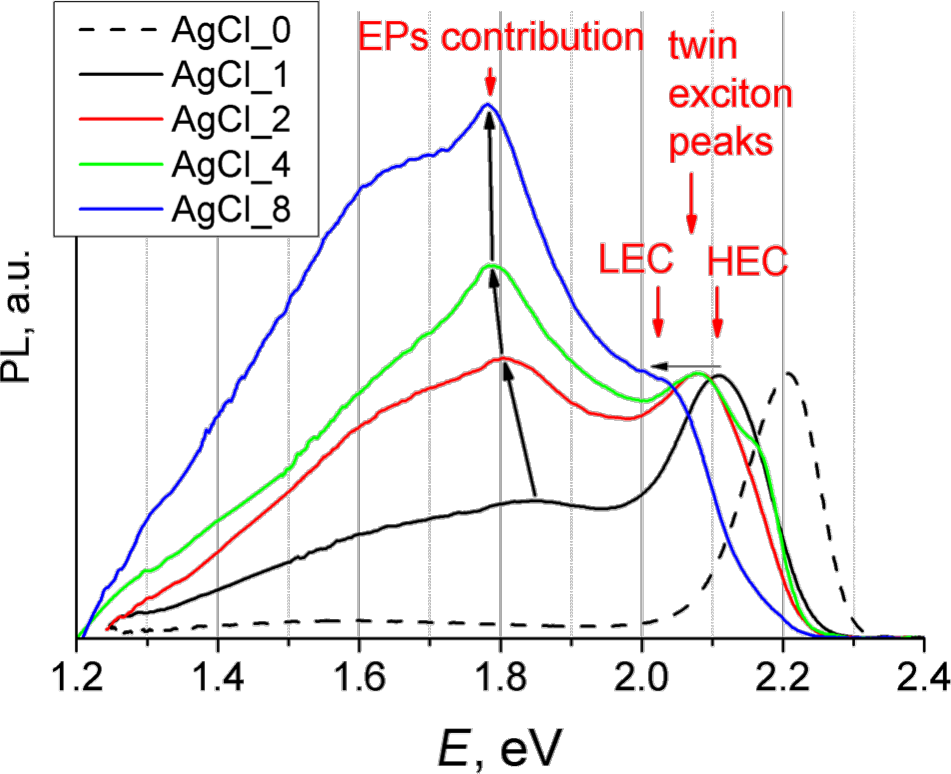
PL spectra of the samples AgCl_0, AgCl_1, AgCl_2, AgCl_4 and AgCl_8.

The typical PL spectrum of undoped NPs (Figure 6, black dotted line) consists of two peaks, an exciton peak (2.21 eV) and a weak wide peak in the low-energy region (1.60 eV) (see semi-logarithmic plot in Supporting Information File 1, Figure S3), which is associated with structure defects. An addition of AgCl precursor greatly affects the PL spectra of synthesized CdSe NPs. The high complexity of the PL spectra of doped NPs presented in Figure 6 is due to a large number of different types of NPs (different shape, size, amount of doping atoms).

The first element of these spectra is the twin exciton peak in the lower (than for undoped NPs) energy region (Figure 6, 2.05–2.20 eV), which is associated with exciton recombination in TPs [28]. The exciton peak contains two components: a low-energy component (LEC, 2.05–2.10 eV) and a high-energy component (HEC, 2.10–2.20 eV). The components are clearly seen for sample AgCl_4 in Figure 6 (green line). The ratio between those two components changes with the amount of AgCl added: HEC prevails at short leg lengths (for Sample AgCl_1, Figure 6, black line) and LEC prevails at higher leg lengths (for Sample AgCl_8, Figure 6, blue line).

The second element is a complex wide peak (WP) in the low-energy region (Figure 6, with maxima at 1.60–1.85 eV). The relative intensity of WP to HEC/LEC increases with the amount of AgCl during synthesis up to sample AgCl_8.

**Figure 7:**
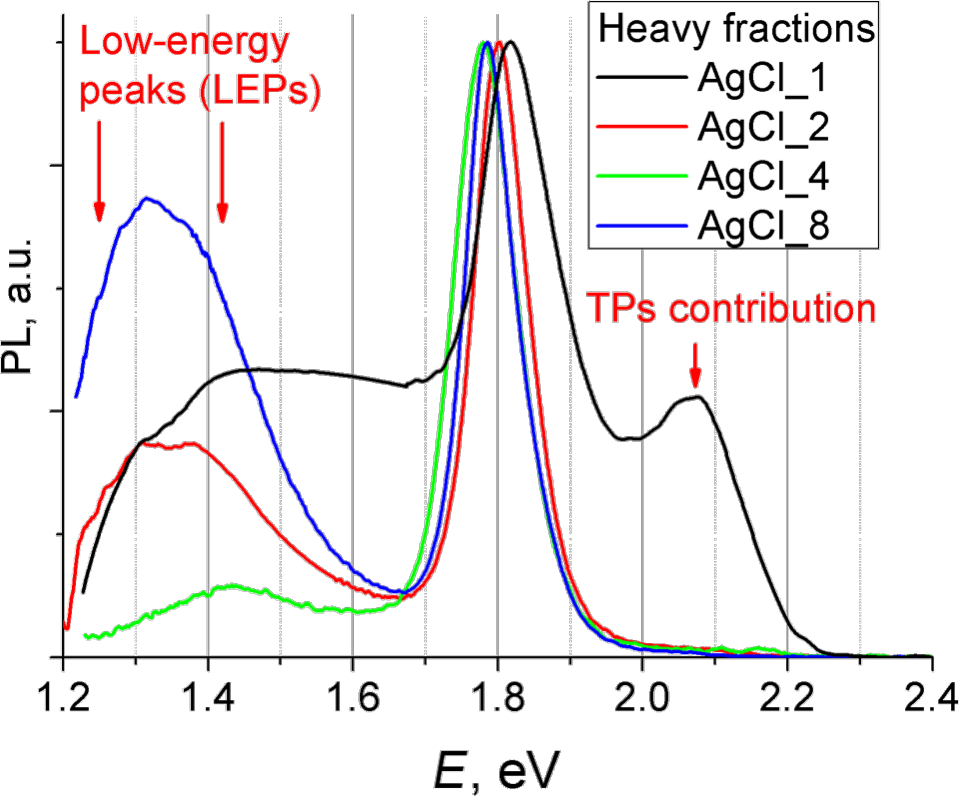
PL spectra of heavy fractions of samples AgCl_1, AgCl_2, AgCl_4 and AgCl_8.

Samples AgCl_1 to AgCl_8 contain two types of particles and both of them should make their contribution to PL spectra. Hence, an attempt was made to separate TPs and EPs. The comparison of the volume of TPs and EPs (Table 2) leads to the conclusion that TPs possess lower mass than EPs and the latter (heavy fraction) can be easily separated from the former (light fraction) from hexane sols with the use of a high-speed centrifuge (21000*g*). The details of this separation are presented in Supporting Information File 1 (the procedure of separation between different fractions). PL spectra of the heavy fractions are presented in Figure 7. It was observed that their PL extends deep in the low-energy region (near-IR region). It is necessary to mention that the numerical proportion of heavy fraction in sample AgCl_1 is minimal. Therefore it was difficult to fully separate this fraction (see Figure 7, black line). The WP component of TPs further complicates the interpretation of the spectrum.

**Figure 8:**
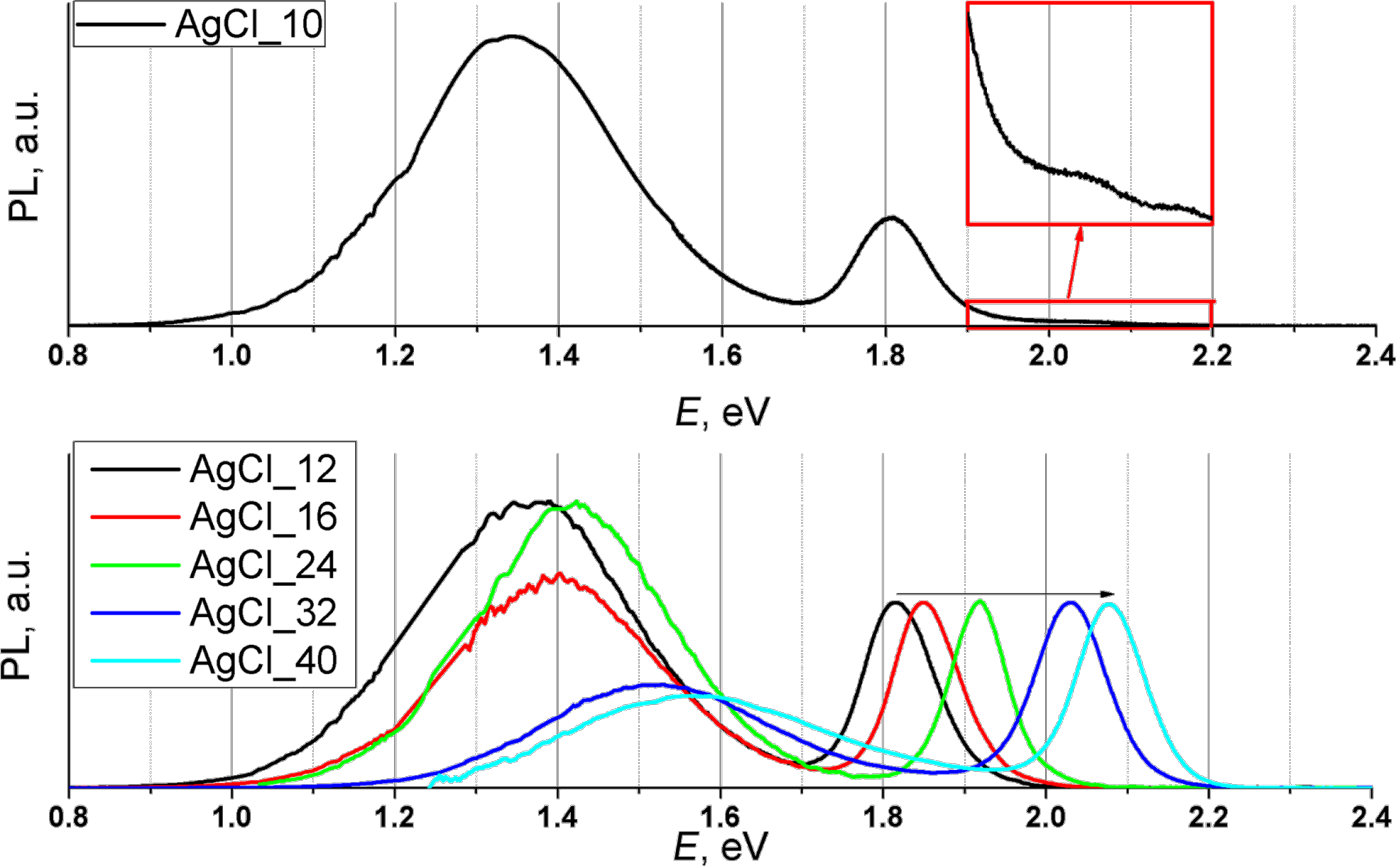
PL spectra of samples AgCl_10, AgCl_12, AgCl_16, AgCl_24, AgCl_32 and AgCl_40.

Figure 8 shows PL spectra of samples AgCl_10 to AgCl_40. These samples (concept for sample AgCl_10) do not contain TPs. Thus, their PL spectra belong to EPs. PL spectrum of sample AgCl_10 contains three exciton bands, at 2.16, 2.04, and 1.80 eV, which correspond to exciton recombination in TPs (the first two) and in large EPs, respectively. The increase of the energy of the third exciton peak (1.78 to 2.05 eV) in samples AgCl_10 to AgCl_40 is due to the size reduction of EPs (Table 2). A monotonic dependence of the low-energy peak (LEPs) intensity was not observed.

**Figure 9:**
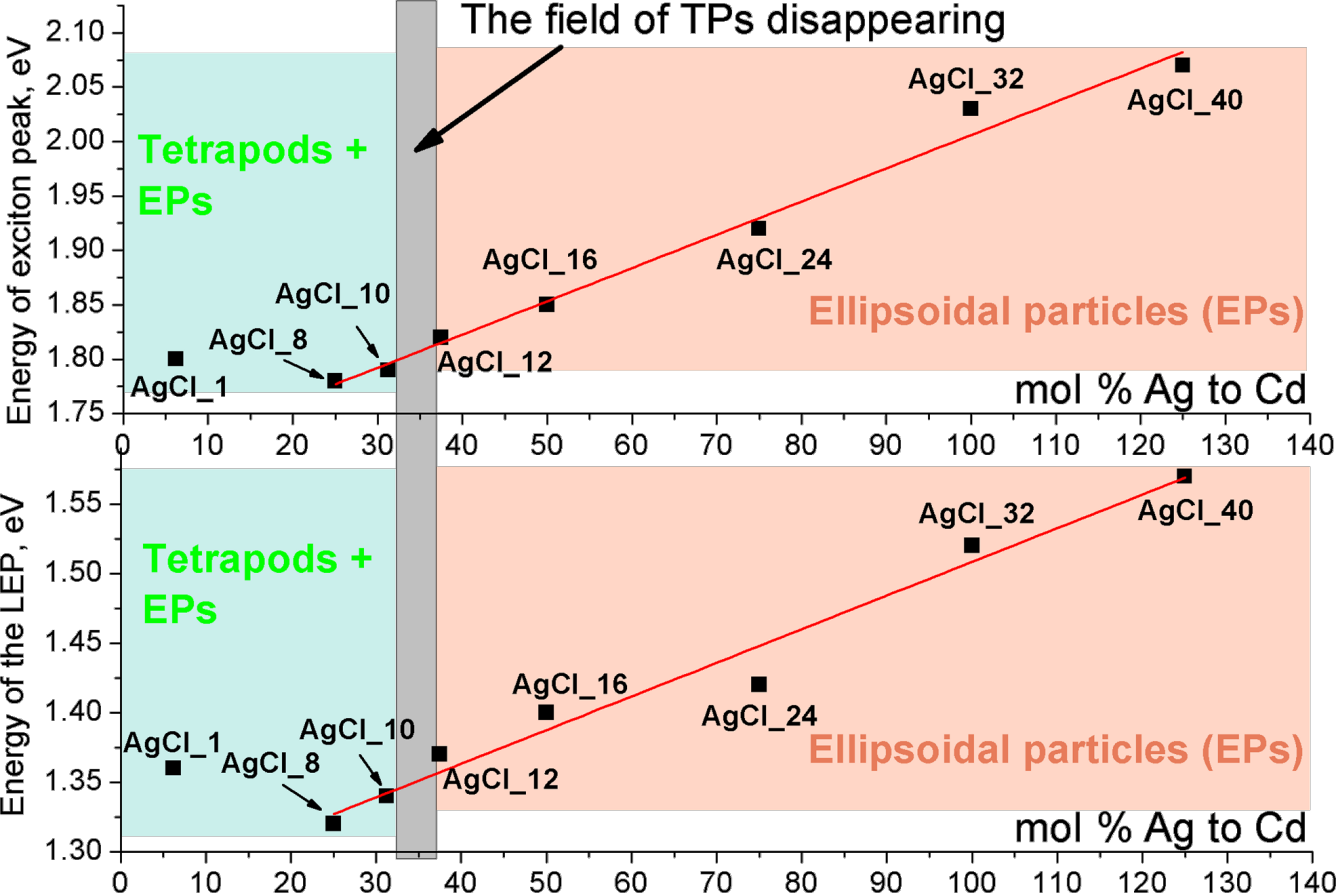
Positions of exciton bands and LEPs maxima from the amount of AgCl added for all samples.

As was mentioned above IR-PL is of great practical interest. In our case EPs play the major role in the formation of near-IR PL. To find the optimal conditions for obtaining near-IR luminescence we compare PL data of EPs from heavy fractions of samples AgCl_1 to AgCl_8 and of samples AgCl_10 to AgCl_40 (see Supporting Information File 1, Table S1). Figure 9 shows the positions of exciton bands and the maxima of LEPs as a function of the amount of AgCl added. On the one hand, one can see that the addition of less AgCl makes it possible to obtain QDs with near-IR-PL. On the other hand, if we take into account the share of EPs in samples (see Table 2), the optimal amount of AgCl in synthesis to get a large number of IR-PL particles is to be about 35 mol % Ag to Cd (see Figure 9, gray rectangle).

**Figure 10:**
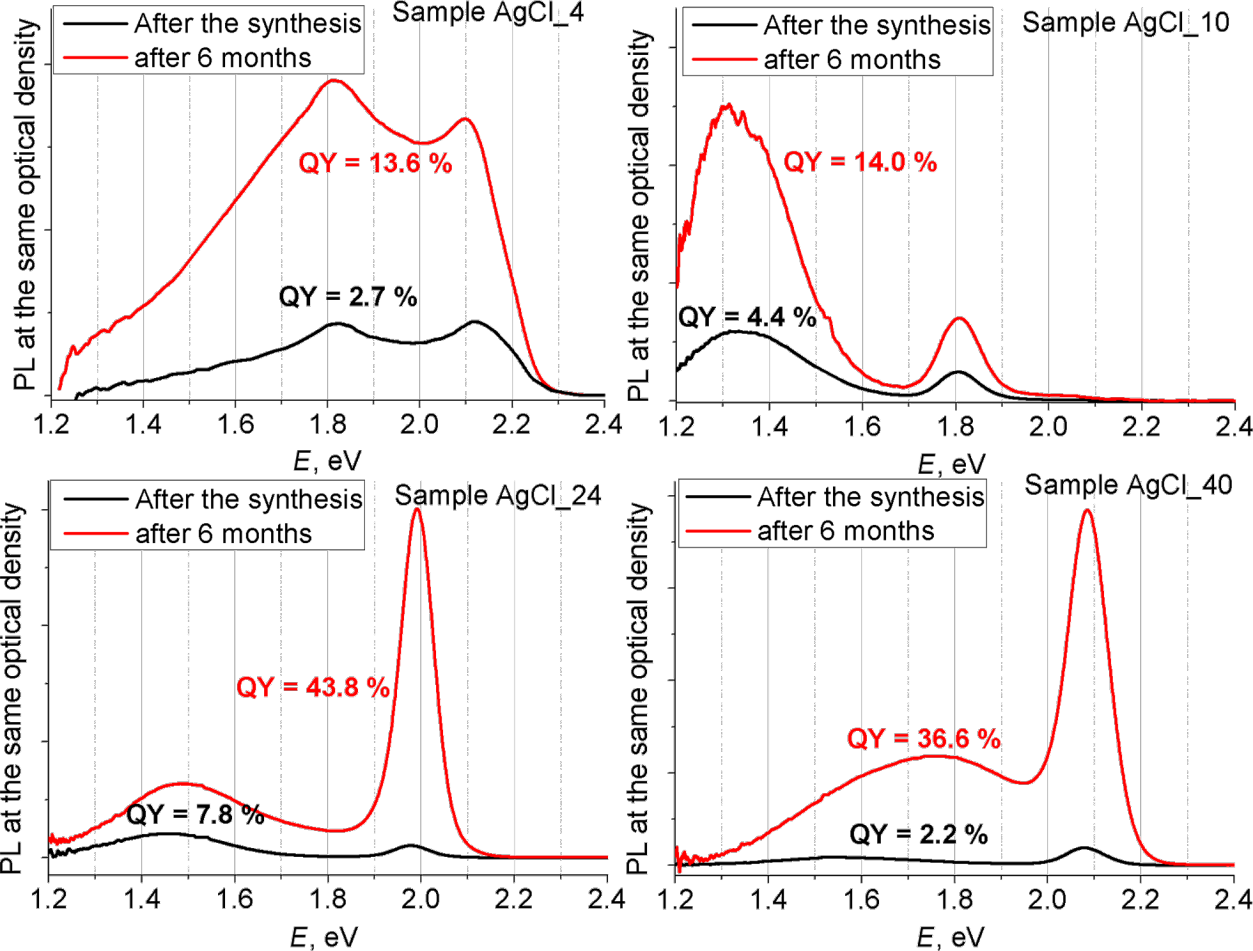
QY values and PL spectra of samples AgCl_4, AgCl_10, AgCl_24 and AgCl_40 after the synthesis immediately and after 6 months.

It was established that the intensity of PL spectra (both exciton and EPs) significantly increases over time (see Figure 10). Values of QY that were obtained directly after the synthesis were lower than 10%. The possible explanation of poor exciton intensity could be the presence of a large number of volume Ag exciton traps. After a long time Ag leaves the NPs and the number of Ag exciton traps is greatly reduced.

At the same time the intensity of the low-energy band also increases. We propose that the reason for the appearance of IR peaks is the high defectiveness of the structure of EPs, which is the result of Ag doping. The following exclusion of Ag leads to even more defective structures and to an increase of the LEP intensity.

**Figure 11:**
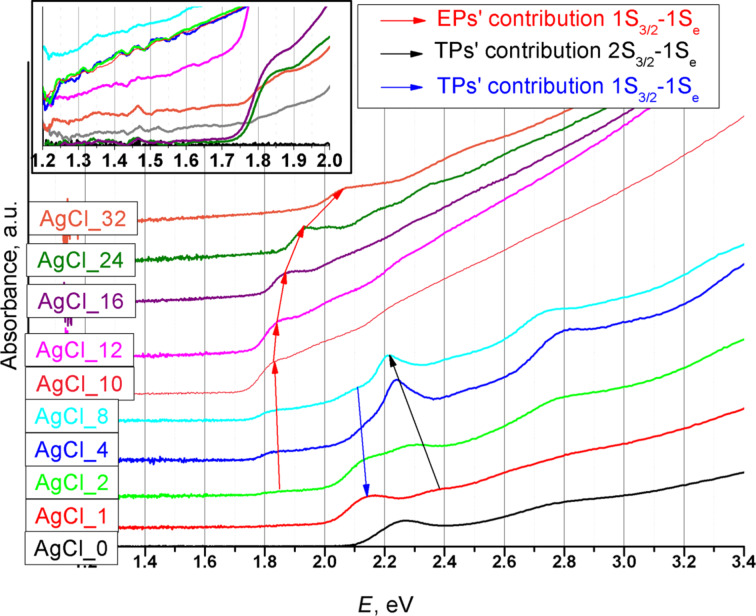
Absorbance spectra of all AgCl-doped CdSe QDs samples. Inset: low-energy regions of spectra without normalization. Legend: assignation of electron transitions according to [28].

The absorbance of the samples illustrates all electron transitions in NPs (Figure 11) [29]. The gradual alteration of the types of NPs (TPs, EPs) causes appearance of new electron transitions and disappearance of others. The red arrow in Figure 11 shows the evolution of 1S_3/2_–1S_e_ electron transition in EPs and spherical particles doped with AgCl. The absorption spectra of TPs begin at 2.0 eV. The black arrow in Figure 11 shows an increase in the intensity of the 2S_3/2_-1S_e_ transition against the background of 1S_3/2_-1S_e_ (blue arrow), which is connected with the increase of legs of TPs [30].

Low-energy regions of the spectra prior to intensity normalization are presented in the inset of Figure 11. Some of the samples possess tails, which can be associated with structure defects. We found that a band tail is an essential attribute of EPs with the low-energy exciton (Supporting Information File 1, Figure S4).

Adding silver along with cadmium and selenium during synthesis obliges us to discuss the possibility of the formation of Ag_2_Se NPs. According to previous investigations, CdSe nanocrystals that were exposed to Ag cations in solution could be completely or partially converted to Ag_2_Se via cation exchange [24]. Also, the possibility of Ag_2_Se formation from Ag^+^ and TOPSe solutions at 125–185 °C was shown [31–32]. The authors of [32] synthesized NPs with an exciton-PL maximum at ca. 1000 nm (ca. 1.24 eV) and sufficiently high QY.

The EPs synthesized in our present work exhibit broad IR-PL exactly at 1.24 eV. However, the *E*_g_ value of bulk Ag_2_Se is rather small, so the excitation-band maximum position at 1.24 eV would correspond to NPs with a mean diameter of 3 nm [32]. Such small NPs are poorly deposited by centrifuging in the absence of a coagulator and therefore should primarily be found in the light fraction. In our work broad IR-PL was found only in the heavy fraction. Moreover, the reduction of the silver concentration down to ca. 1 mol % (to Cd) during sample storage does not affect the form of the PL spectra, while the PL intensity at 1.24 eV even increases. Also, we showed the absence of Ag_2_Se in sols of the samples after the post-synthetic treatment even at high Ag concentrations with the use of EDX-TEM and HRTEM.

At the same time, a closer look at the XRD patterns shows that in the 2θ region of 30–45°, a novel phase with weak diffraction intensity appears when additional AgCl is added to the nanocrystals. This is exactly the region, in which Ag_2_Se shows its most intense Bragg peaks [31]. From these data it can be concluded that Ag_2_Se was formed during the XRD measurements as a product of the photodegradation of CdSe–Ag NPs through the X-ray irradiation.

Together these facts point unequivocally to the absence of a significant contribution of Ag_2_Se to the PL spectra. Also, we assume that Ag_2_Se NPs could be formed in our case, but they were separated from our samples in the post-synthetic stage due to their low colloidal stability [31].

## Conclusion

In our work we investigated CdSe–AgCl nanoparticles that were synthesized by the oleate colloidal method. Here, we found optimal conditions in which IR-PL can be obtained. The most optimal Ag to Cd ratio in synthesis is about 3:8, where the largest ellipsoidal NPs were fabricated. The IR-PL of ellipsoidal NPs, ranging up to 0.9 eV (ca. 1400 nm), is associated with defects present in these NPs.

Our current study showed that the replacement of Ag_4_Cl_4_(PPh_3_)_4_ (which had been used in our previous researches [22,25]) with a AgCl solution in TOP led to a high Ag to Cd ratio in the reaction mass. This made it possible to synthesize NPs with different shapes and strong IR-PL. As was already mentioned, it is mainly related to the low solubility of Ag_4_Cl_4_(PPh_3_)_4_ in the reaction mass. This leads to a low concentration of silver and chloride ions. Hence, only tetrapod-like particles with short legs (about 5 nm) were formed when Ag_4_Cl_4_(PPh_3_)_4_ was added to Cd(oleate)_2_ at a ratio of 1:4 (Ag/Cd = 1:1) [25]. Similar NPs could be obtained with a ratio between AgCl/TOP and Cd(oleate)_2_ of about 1:16 (Ag/Cd = 1:16).

Element distribution maps showed that Ag and Cl are present in QDs (EDX-TEM analysis was performed within one day after the synthesis). XRD and HRTEM measurements confirmed the presence of cubic-phase undoped nanoparticles. Also, there is an increase in the amount of the hexagonal phase in NPs when the AgCl concentration is increased during synthesis. Hexagonal-phase NPs were obtained at the highest AgCl concentrations (Ag to Cd ratios above 1:1 during synthesis).

The particle morphology was also controlled by the amount of AgCl during synthesis. The addition of AgCl allowed us to obtain tetrapods and large ellipsoidal NPs. Tetrapods have a cubic-phase core and hexagonal-phase legs. Large ellipsoidal NPs are mostly diphasic. In our work we assume that large ellipsoidal NPs were formed from the tetrapods by accretion of legs on a core. It was established by the investigation of the so-called “match head” particles, which are an intermediate form between tetrapods and ellipsoidal NPs.

The sample storage was accompanied by the formation of a mixture of Ag and Ag_2_Se, which positively affects the QY of the QDs (from below 10% to ca. 40%) and has only minor effect on the form of the PL spectra.

## Experimental

QDs with different Ag/Cd ratios were synthesized with an oleate method using in situ colloidal synthesis. We used cadmium oleate (prepared beforehand from cadmium acetate and oleic acid) dissolved in diphenyl ether (0.088 M solution) as the cadmium source. Trioctylphosphine selenide (TOPSe, 1 M solution in trioctylphosphine (TOP)) was used as selenium precursor (prepared by dissolving selenium powder in TOP). The Ag precursor was AgCl solution in TOP (0.5 mL). Origin and purity of the reagents are given in Supporting Information File 1.

Each synthesis was performed with the use of the typical procedure described in [22]: The cadmium precursor solution (0.5 mmol) was put into a flask together with 0.5 mL AgCl solution (the quantities of AgCl for each synthesis are given in Table 1). Afterwards, the obtained mixture was heated to 200 °C in Ar flow, and 0.45 mL of 1 M TOPSe solution in TOP was injected while stirring. After particle growth (for 5 min), the reaction system was cooled down to room temperature. It is to be noted that the rate of colour alteration strongly depends on the amount of AgCl added. Typically, 10–30 sec after injection the solution of undoped QDs changed from pale yellow to yellow and then to red, while, adding 500 μmol of AgCl led to an alteration of colour in 2–3 sec. For the post-synthetic purification an equal amount of acetone used as an anti-solvent was added to the samples, resulting in the precipitation of QDs. The precipitated QDs were redispersed in hexane. Then the QDs were precipitated with acetone and dispersed in hexane again to achieve sufficient purity. The samples dispersed in hexane were stored in glass vials in dark.

The elemental analysis was done with a Bruker Mistral M1 μ-XRF spectrometer. The Ag to Cd ratio was determined from the relative intensities of the Kα_1_ peaks with the use of a calibration curve (Supplementary data of [22]). UV–vis absorption measurements were carried out with a Varian Cary 50 spectrophotometer from 300 to 1100 nm. The fluorescence was measured with 405 nm laser excitation and detected by an Ocean Optics USB 4000 spectrometer calibrated by a 2600 K W-lamp (450–1100 nm). The energy QYs were determined in diluted dispersions relative to rhodamine 6G solution of the same absorbance at 405 nm. IR-photoluminescence spectra were obtained with a MDR-23 monochromator with Ge photodetector calibrated with a 2600 K W-lamp.

Transmission electron microscopy (TEM) studies were performed using JEOL JEM2100 and Tecnai G2 30 UT (LaB_6_) microscopes operated at 200 and 300 kV, respectively. The latter has 0.17 nm point resolution and is equipped with an EDAX EDX detector. High-angle annular dark field (HAADF) STEM studies and EDX mapping were performed using an JEM ARM200F cold FEG double aberration-corrected electron microscope operated at 80 kV and equipped with a large solid-angle CENTURIO EDX detector and Quantum EELS spectrometer. TEM samples were obtained by drop-casting QD/hexane sols on Cu holey and formvar carbon grids. Size distributions were obtained from TEM images. XRD measurements were performed with the use of a Rigaku D/MAX 2500 (Cu Kα radiation, rotating anode, Bragg–Brentano scheme, graphite monochromator) diffractometer.

## Supporting Information

File 1 Additional figures, data and experimental information.

## References

[1] Alivisatos A P (1996). Science.

[2] Hoy J, Morrison P J, Steinberg L K, Buhro W E, Loomis R A (2013). J Phys Chem Lett.

[3] Luther J M, Pietryga J M (2013). ACS Nano.

[4] de Mello Donegá C, Koole R (2009). J Phys Chem C.

[5] Landry M L, Morrell T E, Karagounis T K, Hsia C-H, Wang C-Y (2014). J Chem Educ.

[6] Jasieniak J, Smith L, van Embden J, Mulvaney P (2009). J Phys Chem C.

[7] Groeneveld E, Delerue C, Allan G, Niquet Y-M, de Mello Donegá C (2012). J Phys Chem C.

[8] Kamal J S, Omari A, Van Hoecke K, Zhao Q, Vantomme A, Vanhaecke F, Capek R K, Hens Z (2012). J Phys Chem C.

[9] Mićić O I, Cheong H M, Fu H, Zunger A, Sprague J R, Mascarenhas A, Nozik A J (1997). J Phys Chem B.

[10] Van de Walle C G, Neugebauer J (2003). Nature.

[11] Wang Q, Seo D-K (2006). Chem Mater.

[12] Nann T, Skinner W M (2011). ACS Nano.

[13] Califano M, Gomez-Campos F M (2013). Nano Lett.

[14] Abdellah M, Karki K J, Lenngren N, Zheng K, Pascher T, Yartsev A, Pullerits T (2014). J Phys Chem C.

[15] Tyagi P, Kambhampati P (2011). J Chem Phys.

[16] Kobayashi Y, Nishimura T, Yamaguchi H, Tamai N (2011). J Phys Chem Lett.

[17] Norris D J, Efros A L, Erwin S C (2008). Science.

[18] Whitham P J, Knowles K E, Reid P J, Gamelin D R (2015). Nano Lett.

[19] Pradhan N, Goorskey D, Thessing J, Peng X (2005). J Am Chem Soc.

[20] Meulenberg R W, van Buuren T, Hanif K M, Willey T M, Strouse G F, Terminello L J (2004). Nano Lett.

[21] Hamanaka Y, Ozawa K, Kuzuya T (2014). J Phys Chem C.

[22] Bubenov S S, Dorofeev S G, Kotin P A, Znamenkov K O, Kuznetsova T A (2014). Mendeleev Commun.

[23] Sahu A, Kang M S, Kompch A, Notthoff C, Wills A W, Deng D, Winterer M, Frisbie C D, Norris D J (2012). Nano Lett.

[24] Son D H, Hughes S M, Yin Y, Alivisatos A P (2004). Science.

[25] Kotin P A, Bubenov S S, Kuznetsova T A, Dorofeev S G (2015). Mendeleev Commun.

[26] Greaney M J, Couderc E, Zhao J, Nail B A, Mecklenburg M, Thornbury W, Osterloh F E, Bradforth S E, Brutchey R L (2015). Chem Mater.

[27] Lim J, Bae W K, Park K U, zur Borg L, Zentel R, Lee S, Char K (2013). Chem Mater.

[28] Nghia N X, Hai L B, Luyen N T, Nga P T, Thuy Lieu N T, Phan T-L (2012). J Phys Chem C.

[29] Ekimov A I, Hache F, Schanne-Klein M C, Ricard D, Flytzanis C, Kudryavtsev I A, Yazeva T V, Rodina A V, Efros Al L (1993). J Opt Soc Am B.

[30] Mohamed M B, Tonti D, Salman A A, Chergui M (2005). ChemPhysChem.

[31] Sahu A, Qi L, Kang M S, Deng D, Norris D J (2011). J Am Chem Soc.

[32] Shi L-J, Zhu C-N, He H, Zhu D-L, Zhang Z-L, Pang D-W, Tian Z-Q (2016). RSC Adv.

