# Supplementary material for: AgCl-doped CdSe quantum dots with near-IR photoluminescence
Source: Beilstein J Nanotechnol. 2017 May 29;8:1156–66. doi: 10.3762/bjnano.8.117 (PMC5480357; doi:10.3762/bjnano.8.117)
Supplement: File 1 — Additional figures, data and experimental information. [file Beilstein_J_Nanotechnol-08-1156-s001.pdf]

Supporting Information

for

AgCl-doped CdSe quantum dots with near-IR photoluminescence

Pavel A. Kotin*[1], Sergey S. Bubenov[1], Natalia E. Mordvinova[1,2] and Sergey G. Dorofeev[1]

Address: [1]Department of Chemistry, Lomonosov Moscow State University, 1 building 3 Leninskie Gory, Moscow 119991, Russia and [2]Laboratoire CRISMAT, UMR6508, CNRS-ENSICAEN, 6 boulevard Marechal Juin, Caen 14050, France

Email: Pavel Aleksandrovich Kotin* - kotin-pa@mail.ru

* Corresponding author

Additional figures, data and experimental information

Additional TEM images of samples

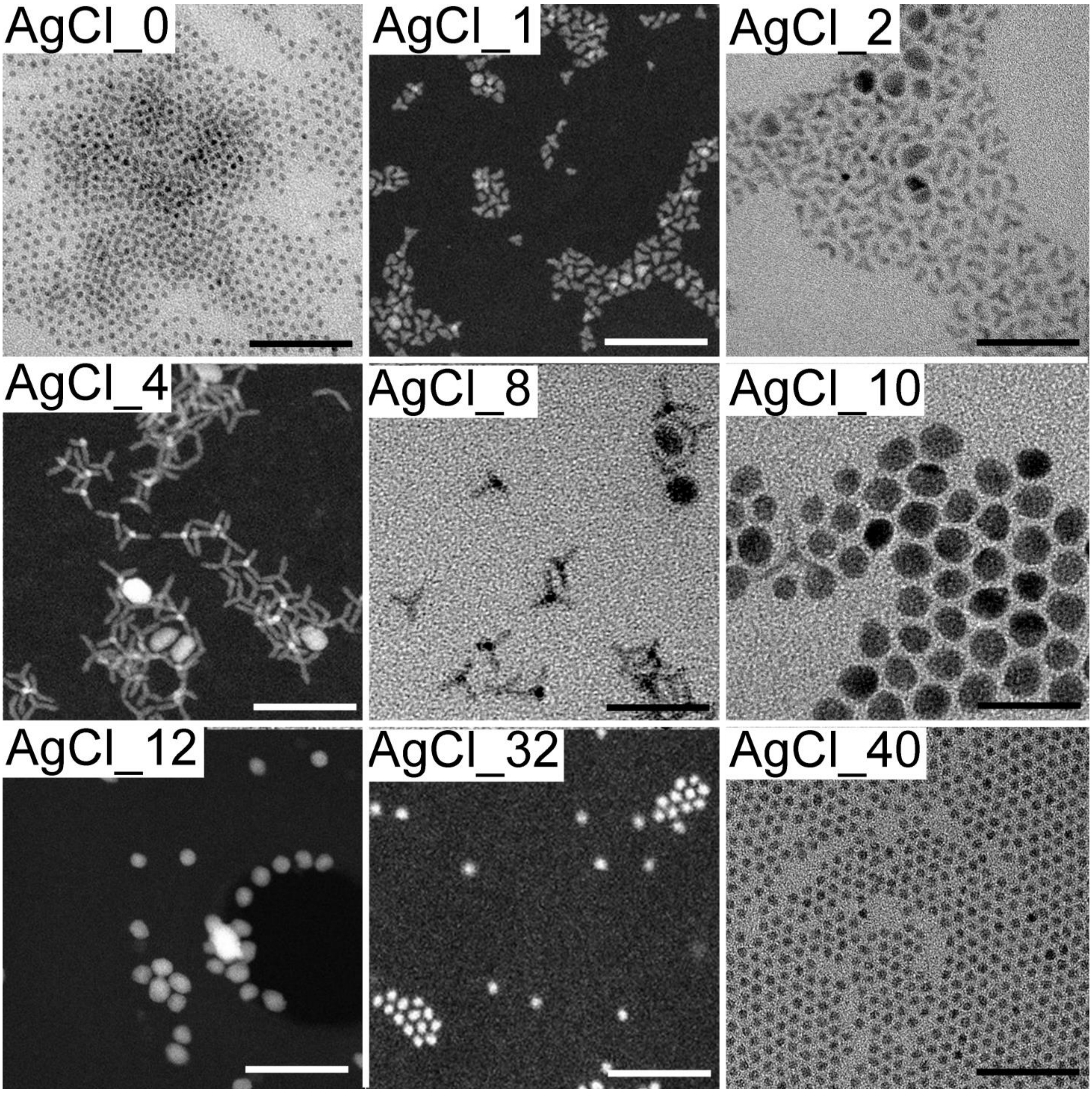

Figure S1: TEM images of samples AgCl_0 to AgCl_40. The scale bar is 50 nm.

Additional XRD pattern

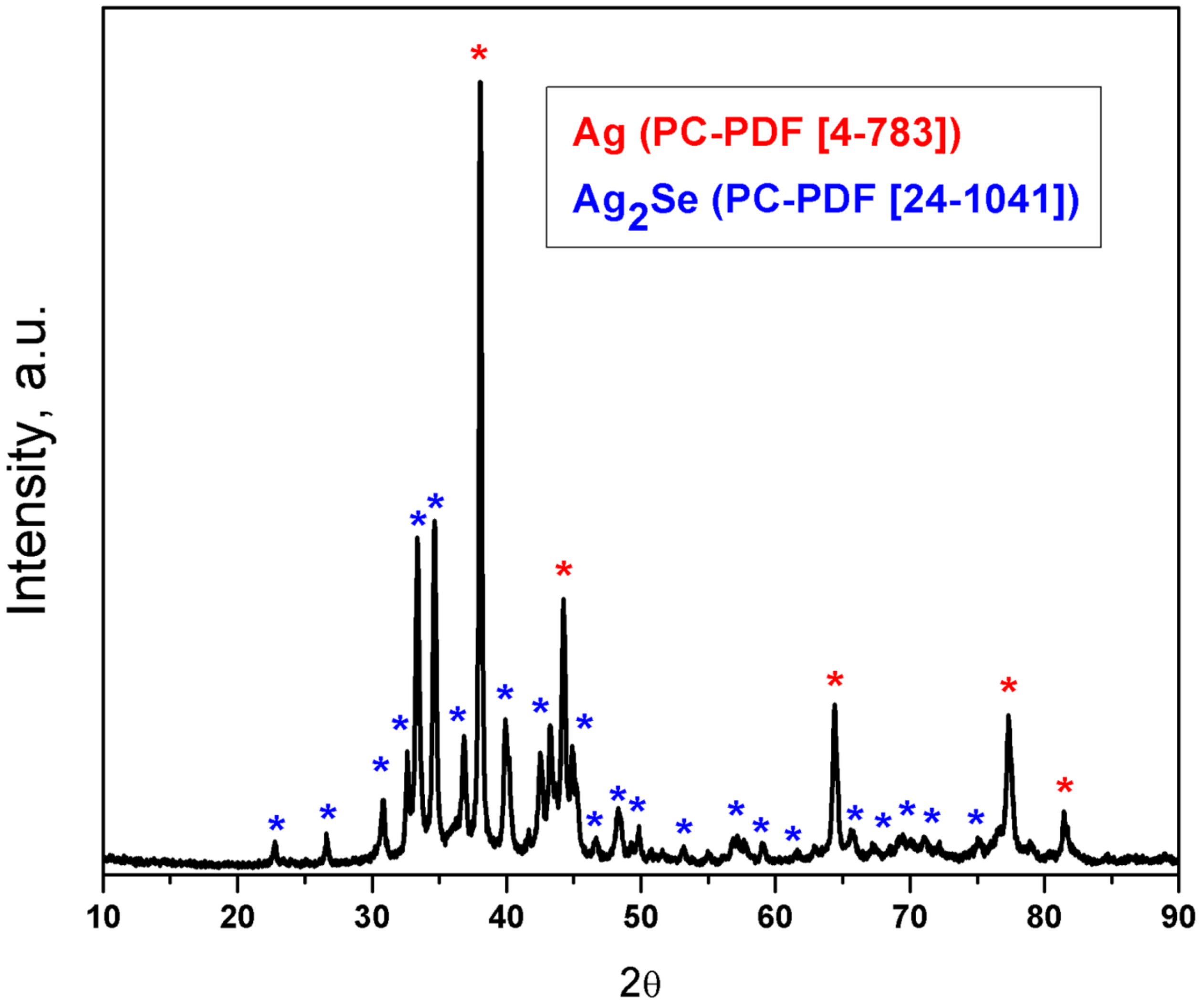

Figure S2: XRD pattern of deposition from the Sample AgCl_40 during the storage.

Additional optical data

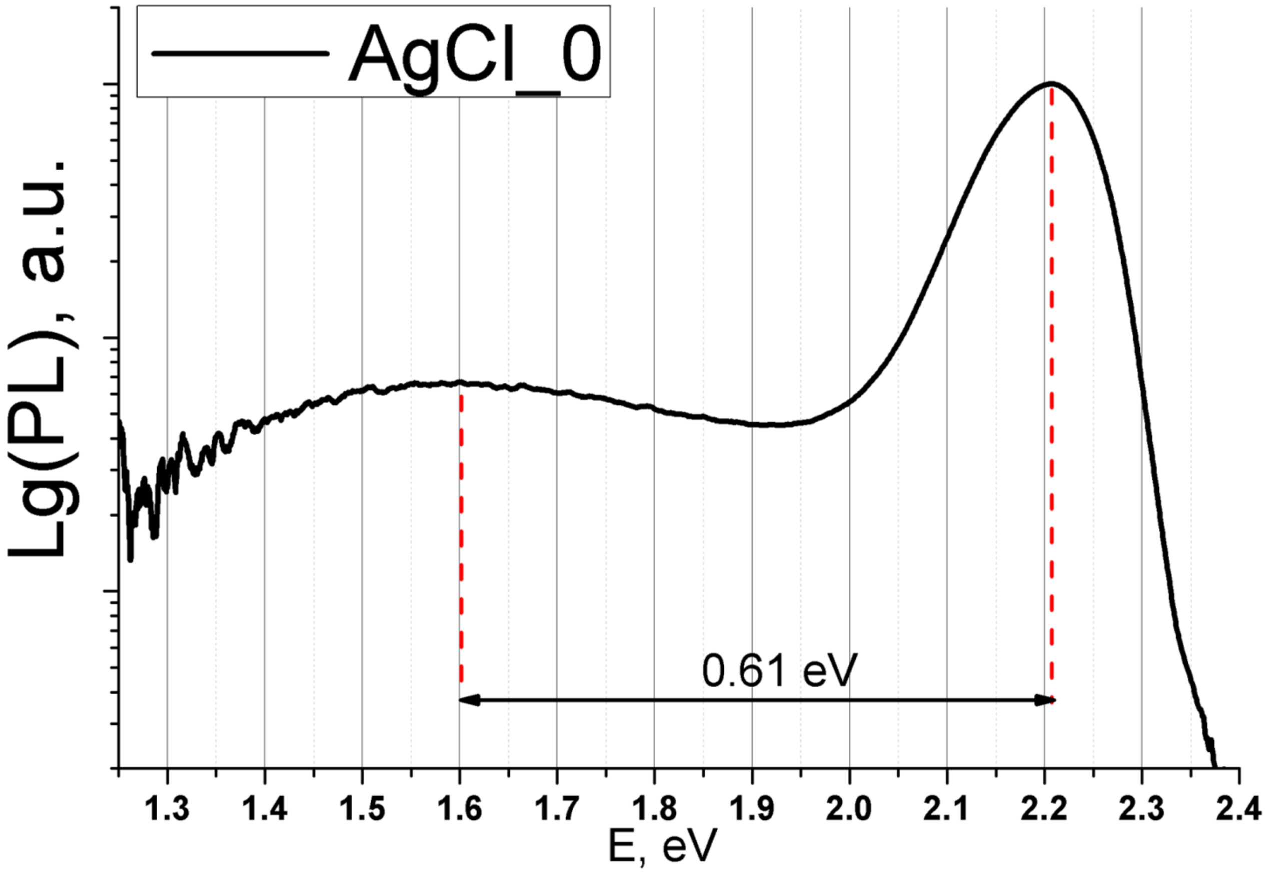

Figure S3: Semi-log plot of PL spectrum of undoped CdSe QDs.

Table S1: Positions of exciton bands, LEPs and energy difference between them.

Sample	Exciton, eV	LEP, eV	Δ*E*, eV
AgCl_0	2.21	1.6 (spherical)	0.61
AgCl_1	1.82	—	—
AgCl_2	1.80	1.36	0.42
AgCl_4	1.78	1.44	0.34
AgCl_8	1.78	1.34	0.44
AgCl_10	1.79	1.34	0.45

AgCl_12	1.82	1.36	0.46
AgCl_16	1.85	1.40	0.45
AgCl_24	1.92	1.42	0.50
AgCl_32	2.03	1.52	0.51
AgCl_40	2.07	1.57	0.50

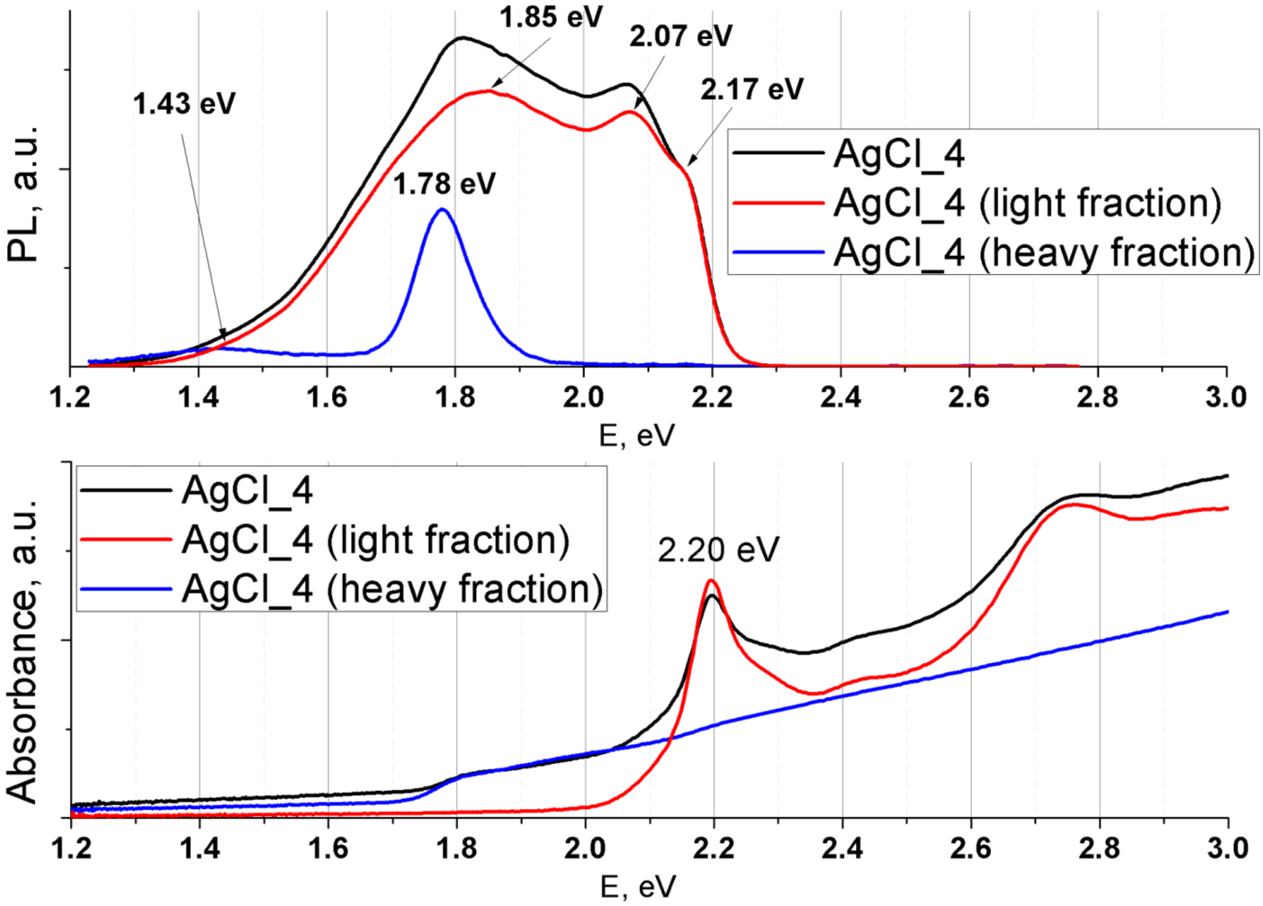

Figure S4: PL spectra (top) and absorbance (bottom) of different fractions of sample AgCl_4.

The procedure of separation between different fractions

Separation of TPs and EPs was performed by centrifugation in a high-speed centrifuge (21000*g*). We refer to EPs as heavy fraction and to TPs as light fraction. Slow deposition of TPs is due to higher hydrodynamic size-mass ratio and larger surface area (which results in a larger number of ligands and increased solubility) than that of EPs.

The typical procedure of isolation of heavy fraction implies a number of 5 min centrifugations. The fraction was selected from the bottom of centrifuge tube. A series of short time acts is required to prevent the coprecipitation of light fraction. Isolation of EPs from the sample AgCl_1 required five iterations of 5 min centrifuging, for the Samples AgCl_2, AgCl_4, AgCl_8, AgCl_10 it required four, four, two and one iterations of 5 minute centrifuging respectively.

The procedure of isolation of light fraction implies three 30 min centrifugations. The fraction was selected from the top of the centrifuge tube. A long time is required to fully remove the heavy fraction.

Purity of reagents

The starting chemicals used were cadmium acetate, $Cd(CH_3COO)_2{\cdot}2H2O$ (analytical-grade); silver chloride, AgCl (analytical-grade); oleic acid (Fluke, 95%); trioctylphosphine (TOP) (Aldrich, 90%); selenium (extrapure grade); acetone, hexane and dodecane (extrapure grade); diphenyl ether (abcr, 99%).
